# The Impact of Cholesterol, DHA, and Sphingolipids on Alzheimer's Disease

**DOI:** 10.1155/2013/814390

**Published:** 2014-02-19

**Authors:** Marcus O. W. Grimm, Valerie C. Zimmer, Johannes Lehmann, Heike S. Grimm, Tobias Hartmann

**Affiliations:** ^1^Experimental Neurology, Saarland University, Kirrberger Street 1, 66421 Homburgr, Saar, Germany; ^2^Neurodegeneration and Neurobiology, Saarland University, Kirrberger Street 1, 66421 Homburg, Germany; ^3^Deutsches Institut für DemenzPrävention (DIDP), Saarland University, Kirrberger Street 1, 66421 Homburgr, Saar, Germany

## Abstract

Alzheimer's disease (AD) is a devastating neurodegenerative disorder currently affecting over 35 million people worldwide. Pathological hallmarks of AD are massive amyloidosis, extracellular senile plaques, and intracellular neurofibrillary tangles accompanied by an excessive loss of synapses. Major constituents of senile plaques are 40–42 amino acid long peptides termed **β**-amyloid (A**β**). A**β** is produced by sequential proteolytic processing of the amyloid precursor protein (APP). APP processing and A**β** production have been one of the central scopes in AD research in the past. In the last years, lipids and lipid-related issues are more frequently discussed to contribute to the AD pathogenesis. This review summarizes lipid alterations found in AD *postmortem* brains, AD transgenic mouse models, and the current understanding of how lipids influence the molecular mechanisms leading to AD and A**β** generation, focusing especially on cholesterol, docosahexaenoic acid (DHA), and sphingolipids/glycosphingolipids.

## 1. APP Processing

Amyloid plaques are composed of aggregated amyloid-*β* peptides, derived from sequential proteolytic processing of the amyloid-precursor protein (APP), a type-I transmembrane protein with a large extracellular N-terminal domain and a short intracellular C-terminal tail [1]. APP and its gene family members, the APP-like proteins APLP1 and APLP2, are highly conserved proteins expressed in numerous species and tissues pointing out their physiological importance [2]. Indeed, triple knockout of APP/APLP1/APLP2 in mice results in postnatal lethality involving brain development abnormalities and cortical dysplasia [3]. In contrast, single knockout of APP has a minor phenotype consisting of reduced body weight [2], commissure defects [4], and hypersensitivity to epileptic seizures [5] demonstrating the mutual functional compensation of the gene family members and additionally illustrates the physiological role of APP. Furthermore, a potential contribution to the formation of dendritic and synaptic structures as well as in long-term potentiation (LTP) [6–8] suggests a possible impact on cognitive function.

APP can be cleaved in two distinct pathways, the amyloidogenic and nonamyloidogenic pathways. The non-amyloidogenic processing of APP by *α*-secretases avoids the formation of A*β* peptides by cleaving inside the A*β* domain [9]. Thereby, the large N-terminal ectodomain *α*-secreted APP (sAPP*α*) is released into the extracellular matrix, whereas the short C-terminal part (*α*-CTF) remains within the membrane for further processing. Notably, sAPP*α* has neuroprotective and memory enhancing properties [10, 11], hypothesizing that sAPP*α* might mediate a major physiological function of APP [12]. The *α*-secretases belonging to the ADAM family (a disintegrin and metalloprotease), in particular ADAM10 and ADAM17 (tumor necrosis factor *α* converting enzyme/TACE), have emerged as predominant *α*-secretases [13–15]. Like APP itself, these proteins are type I integral membrane proteins.

The amyloidogenic pathway, on the other hand, is initiated by the *β*-secretase BACE1 (beta-site APP cleaving enzyme) that generates soluble *β*-secreted APP (sAPP*β*) and the membrane-tethered fragment *β*-CTF. BACE1 is a membrane-bound aspartyl protease belonging to the pepsin family, expressed in neurons [16]. The main *β*-secretase activity is found in the secretory pathway including the Golgi compartments, secretory vesicles, and endosomes [17, 18]. Apart from its amyloidogenic effect, less is known about the physiological function of BACE1; however, in BACE1 knock-out mice, myelination is affected [19, 20]. Initial cleavage of APP by *α*-secretase in the non-amyloidogenic pathway, or by *β*-secretase in the amyloidogenic pathway, is typically followed by *γ*-secretase processing, a multimeric complex consisting of at least four subunits—presenilin 1 (PS1) or presenilin 2 (PS2), anterior pharynx defective 1 homologue (APH1), presenilin enhancer 2 (PEN2), and nicastrin [21] with PS1/PS2 being the catalytic centre [22, 23]. Importantly, mutations inside the PS genes are responsible for early onset AD [24]. PS1 and PS2 are multitransmembrane spanning aspartyl proteases cleaving the C-terminal stubs *α*- and *β*-CTF within the centre of their transmembrane domain [25], generating p3 and A*β* peptides of varying length (e.g., A*β*38, A*β*40, and A*β*42). Among the A*β* species generated, hydrophobic A*β*42 peptides self-aggregate to small oligomers before being manifested as senile plaques composed of a dense core of amyloid fibrils [26, 27]. Simultaneously, the APP intracellular domain (AICD), which is discussed to regulate gene transcription, is released into the cytosol [28]. The substrate APP and the secretases involved in APP cleavage are all transmembrane proteins, suggesting that the surrounding lipid microenvironment may play a pivotal role in the pathogenesis of the disease.

## 2. Cholesterol and AD

The human brain has a very high cholesterol content, mainly associated with myelin. Due to the limited transport over the blood-brain barrier (BBB), the brain cholesterol level is largely independent of the serum cholesterol concentrations. The vast majority of brain cholesterol is provided by glial *de novo* synthesis. Noteworthy, cholesterol transport between neurons and glia cells is mostly provided by clusterin/apolipoprotein J (ApoJ) and apolipoprotein E (ApoE) containing lipoproteins. Intriguingly, the ApoE*ε*4 allele genotype is the predominant genetic risk factor for AD, whereas the *ε*2 allele seems to be protective and *ε*3 being the most common form. A possible explanation for this might be reduced A*β*-clearance and/or increased formation of amyloid in the presence of the *ε*4 genotype, but many other mechanisms have also been suggested [29–31]. Recent genome-wide association studies (GWASs) have greatly extended our knowledge on AD risk genes. Interestingly, two main functional risk clusters were identified. Most GWAS-identified risk genes are either linked to inflammation or to lipid/membrane processes. Besides ApoE polymorphism, single-nucleotide polymorphisms for clusterin (CLU), ABCA7, and PICALM were discovered to raise the individual risk for developing AD [32–34]. On one hand, clusterin serves as a major lipid binding protein [35]. Interestingly, clustrin mRNA and protein levels are increased in AD, thereby, correlating with disease severity [36, 37]. Furthermore, clusterin may be involved in modulation of apoptosis, inflammation, and A*β* aggregation [38–40]. ABCA7, belonging to the ATP-binding cassette transporters, is expressed throughout the brain and especially in hippocampal neurons [41, 42]. ABCA7 is involved in cholesterol efflux from cells to ApoE and affects APP processing [43]. These genetic links between AD risk and cholesterol are supported by strong epidemiological evidence linking hypercholesterolemia with dementia [44, 45]. Like many other life-style influenced AD risk factors, elevated blood cholesterol levels, even if only moderately increased, are most relevant if present at midlife [46]. Additionally, elevated ^24^OH-cholesterol levels were observed in the serum of AD patients [47].

Cholesterol is an essential component of mammalian cell membranes. Based on its extraordinary structure, consisting of a fused rigid ring system, a polar hydroxyl group, and a hydrocarbon tail, cholesterol is essential for bilayer's function and organisation. Due to the impact of the rigid ring system, cholesterol can increase the order within the membrane and thereby affects membrane fluidity. Especially in lipid microdomains, envisioned as so-called “lipid rafts,” primarily found in the plasma membrane, the trans-Golgi, and endosomal membranes, this feature is extremely important. Lipid rafts are strongly enriched in sphingomyelin, glycosphingolipids, and cholesterol. Cholesterol provides tight packing of the lipids in these microdomains. Some membrane proteins are preferentially sited in these ordered microdomains. Mechanistically important is the transient colocalization of APP with the amyloidogenic secretases, *β*-secretase, and *γ*-secretase, in the lipid rafts, pointing out that within the amyloidogenic pathway the close colocalization of these proteins is, at least partly, mediated by cholesterol [48, 49]. Nonamyloidogenic processing and the *α*-secretases, however, are localized outside the lipid rafts [50]. This characteristic implies the possible regulation of the nonamyloidogenic and amyloidogenic pathways by altered membrane cholesterol amounts. Indeed, evidence suggests that cholesterol tends to form complexes with *β*-CTF and potentially with full-length APP. Thereby, the translocation of *β*-CTF/APP is promoted to the cholesterol-enriched microdomains and thus providing them to the amyloidogenic processing [51, 52]. Additionally, it is well established that cholesterol directly stimulates *β*- and *γ*-secretases; *vice versa* cholesterol depletion by cyclodextrin or statins leads to a decreased activity [53–55]. Recently, it was described that statins influence APP maturation and phosphorylation not by cholesterol lowering but by the loss of cholesterol precursors [56]. Remarkably, cholesterol further affects the thickness of the lipid bilayer which directly influences the *γ*-secretase cleavage activity and specificity. With increasing membrane thickness, smaller A*β* species (e.g., A*β*38 and A*β*40) were preferentially produced, whereas A*β*42/43 only occurred in smaller amounts [57]. Additionally, *α*-secretase cleavage is increased by lowering cholesterol levels. This effect was attributed to increased membrane fluidity and impaired APP internalisation. After treatment with lovastatin, ADAM10 protein level was elevated [50]. Aside from its impact on the A*β* generation, cholesterol might even modify A*β* aggregation and the subsequent neurotoxicity. Under physiological conditions, mainly monomeric A*β* peptides develop from APP processing [58]. Though, in AD these monomers are prone to form oligomers, protofibrils, and fibrils, whereby the oligomers are considered to be the most toxic subspecies [59]. It was highlighted that cholesterol-enriched microdomains promote amyloid aggregation, while depletion of cholesterol leads to reduced aggregation [60]. Recent findings implicated that cholesterol facilitates *β*-sheet formation by direct interaction with Phe19 amino acid of the A*β* peptide [61]. In line with these findings, some studies showed a relationship between elevated cholesterol level and increased toxicity of A*β* oligomers [62, 63]. Contradictory to this, other investigations reported cholesterol to be protective [64, 65]. Hence, although cholesterol is essential for human brain development and function, it can as well be attributed to a potent role in mediating aggregation and neurotoxicity. 

In line with the strong impact of cholesterol on APP processing, there is evidence that the cleavage products of APP themselves affect cholesterol metabolism. In mouse embryonic fibroblasts deficient in APP/APLP2^−/−^ or PS1/2^−/−^ and therefore unable to produce A*β*, cholesterol levels were highly elevated. Interestingly, these cholesterol aberrations were abrogated by treatment with A*β*1-40 displaying a negative feed-back cycle of A*β*1-40 by inhibiting the 3-hydroxy-3-methyl-glutaryl-CoA reductase (HMGCR), the rate-limiting step in cholesterol *de novo* synthesis [66, 67]. Additionally, decreased membrane fluidity and increased cholesterol content in lipid rafts were observed in cells devoid of A*β* [68]. Within the APP family of proteins, APP apparently has an especially important role for cholesterol homeostasis. In APP knock-out mice, cholesterol is strongly increased [66, 69], and APP knockout has a number of additional cholesterol- and lipid-related consequences, including reduced diet-dependent atherosclerosis [70] and increased Niemann-Pick cholesterol phenotype [71]. To some part, this appears to be caused by a lack of AICD, resulting in altered LRP1 levels [72]. LRP1 belongs to a lipoprotein-receptor family mainly involved in the cholesterol uptake of neurons, whereby cholesterol is provided by ApoE-containing lipoproteins [72]. Changes in membrane cholesterol content will trigger homeostatic actions affecting the regulation and membrane content of many other lipids. Accordingly, many other lipids affect the cholesterol regulation and several of these lipids are targeted by APP/A*β* or themselves change A*β* production. 

**Figure 1:**
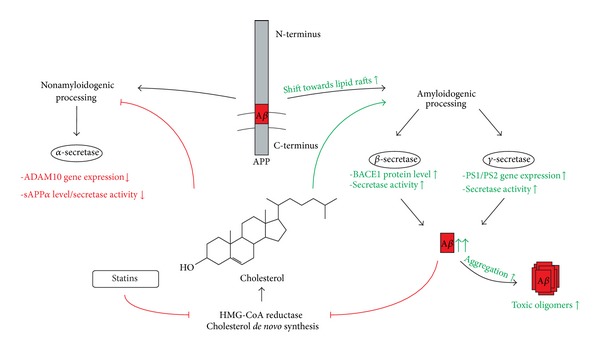
Schematic representation of the proposed mechanisms of cholesterol on APP processing and A*β* aggregation.

Taking all of these findings into consideration (Figure 1; See Table S1 in Supplementary Material available online at http://dx.doi.org/10.1155/2013/814390), pharmacological intervention in cholesterol metabolism might be a possible target in AD treatment. It seems likely that cholesterol lowering by administration of HMGCR inhibitors (e.g., statins) might refer to reduced A*β* levels accompanied by slower cognitive decline and improved mental status.

Animal model studies associated hypercholesteremia with elevated A*β* levels [80–82]. On the opposite, reduced A*β* accumulation was achieved after administration of cholesterol lowering drugs (e.g., statins), underlining their potential role in the disease treatment [83–86]. However, one study reported unaffected A*β* levels, whereas another one even observed increased A*β* deposition [87, 88]. Interestingly, the latter alteration was only detected in female mice. These deviations might be contributed to differences in animal models, used drugs, and period of drug administration. A potential beneficial effect of cholesterol lowering drugs was further investigated in observational studies and randomized controlled trials, whereas many observational studies with high number of participants associated statin intake with reduced development of AD [89–91], others exhibited no differences [92, 93]. However, recent randomized, double-blind, placebo-controlled studies reported no benefit of statin use in individuals suffering from mild to moderate AD [94, 95]. Since in these trials patients were already affected by AD, statins might be ascribed to a more preventive than therapeutic function. Thus, further studies have to evaluate a protective effect in earlier clinical stages.

## 3. Docosahexaenoic Acid and Alzheimer's Disease

Docosahexaenoic acid (DHA) is an essential *ω*-3 polyunsaturated fatty acid (PUFA) mainly found in marine food, especially in oily fish. Only a small amount of DHA can be produced endogenously by synthesis out of *α*-linoleic acid through elongation and desaturation [96], whereas the main DHA is provided by dietary intake. Approximately 60% of the unsaturated fatty acids in neuronal membranes consist of DHA, thus, representing the most common *ω*-3 fatty acid in the human brain. DHA rapidly incorporates into phospholipids of cellular membranes and changes the membrane fluidity by the formation of highly disordered domains concentrated in PUFA-containing phospholipids but depleted in cholesterol (reviewed in [97]). Especially in synapses, these alterations in membrane fluidity play an important role in neurotransmission, ion channel formation, and synaptic plasticity. This suggests a potential role of DHA in memory, learning, and cognitive processes. In young rats, the administration of DHA leads to an improved learning ability [98]. Additionally, DHA is involved in neuronal differentiation [99], neurogenesis [100], and protection against synaptic loss [101]. Therefore, DHA is discussed to be involved in pathological processes of AD. 

DHA is decreased in certain regions of AD *postmortem* brains, like pons, white matter and, in particular, frontal grey matter, and hippocampus [102]. Additionally, peroxidation products of DHA, which is very susceptible to lipid peroxidation due to its six double bounds, are elevated in AD brains hypothesizing that DHA loss can be ascribed to the increased oxidative stress [103]. These lipid alterations seem to occur not only in advanced AD patients but also in early stages of the disease [104].

Several epidemiological studies tried to correlate DHA plasma levels or the amount of dietary intaken fish with cognitive decline. The Rotterdam study presented the first findings about an inverse correlation between increased fish intake and all-cause dementia [105]. However, reexamination with a longer follow-up period found no association between dietary intake of *ω*-3 fatty acids and dementia [106]. Nevertheless, further studies like the PAQUID study [107] or the CHAP study [108] supported the protective effect of fish or DHA consumption initially found in the Rotterdam study. These effects were even more pronounced in individuals not carrying the ApoE *ε*4 allele [109, 110].

Beside the epidemiological studies, findings from animal models and *in vitro* experiments suggest that increased dietary intake of DHA is associated with a reduced risk of AD. In a 3xTg-AD mouse model, that exhibits both A*β* and tau pathologies [111], DHA supplementation caused a significant reduction in soluble and intraneuronal A*β* levels as well as tau phosphorylation [112]. Similar results were obtained with APPSwedish transgenic mice, revealing significant plaque reduction in the hippocampus and parietal cortex accompanied by alterations of APP cleavage products [113]. In AD-model rats, produced by infusion of A*β*1-40 into the cerebral ventricle, DHA also improved learning ability [114]. *In vitro*, A*β* fibrillation was found to be decreased in the presence of DHA [73]. 

Recently, we and others elucidated the molecular mechanisms involved in the reduced A*β* burden in the presence of DHA (summarized in Table 1). In the neuroblastoma cell line SH-SY5Y, DHA directly inhibited amyloidogenic *β*- and *γ*-secretase activities, resulting in a dose-dependent reduction of A*β* levels. On the other hand, non-amyloidogenic APP processing was increased in DHA-treated cells, as we observed elevated sAPP*α* levels caused by an enhanced ADAM17 protein stability [74]. Interestingly, DHA exhibited various interactions with cholesterol homeostasis. Beside a direct inhibition of the HMGCR, DHA induced a cholesterol shift from lipid raft to the nonraft fractions, illustrating an alternative secretase activity modulating pathway [74]. In line with these findings the DHA-mediator NPD1, derived from DHA processing by cytosolic phospholipase A2 and 15-lipooxygenase, is known to directly affect APP processing resulting in elevated sAPP*α* and lower sAPP*β* and accordingly A*β* levels [75, 76]. Interestingly, phospholipase A2 and 15-lipooxygenase and as a consequence NPD1 were found to be reduced in the hippocampus of AD patients and in AD mouse models [75, 76]. Beside the observed shift of APP processing from the amyloidogenic pathway to the non-amyloidogenic pathway, NPD1 also acts neuroprotective by downregulating inflammatory signalling, apoptosis, and A*β*-induced neurotoxicity [76, 115]. 

These findings indicate a possible therapeutic use of DHA in preventing, modulating, or improving AD progression. Clinical trials, however, delivered ambiguous results concerning DHA supplementation and cognitive functions suggesting a limited benefit depending on the disease stage and ApoE allele genotype [116–118]. Further investigations with great cohorts have to clarify whether DHA has more preventing than therapeutic effects by including only patients in the earliest stages of memory decline. More recently, an advanced DHA formulation containing several diet-derived molecules to enhance DHA activity resulted in a number of clinical trials that resulted in various degrees of cognitive benefit. The benefit was most pronounced in patients with very mild to mild AD but less in mild to moderate AD patients [119, 120]. Most recently, at the ADPD conference 2012, Scheltens reported on an open-label extension study in very mild to mild AD patients with continuous increase in memory performance over the study period of 48 weeks [121].

## 4. Sphingo- and Glycosphingolipids

**Figure 2:**
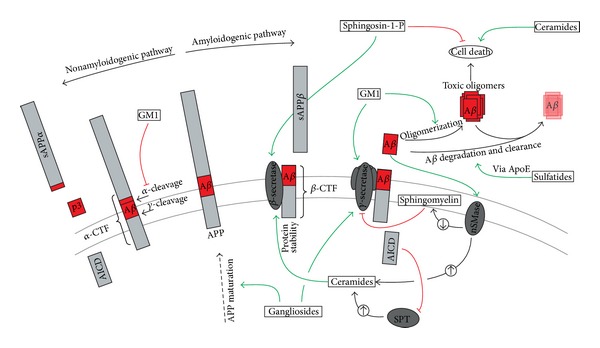
Schematic illustration of the effects of sphingolipids and glycosphingolipids on APP processing. Interestingly, APP processing in return affects the metabolic pathways of sphingolipids. For example, it has been shown that AICD regulates the sphingolipid *de novo* synthesis by decreasing the expression of the Serinepalmitoyl-CoA-Transferase (SPT) or that A*β* itself directly increases the activity of the sphingomyelin degrading enzyme Sphingomyelinase (SMase), resulting in complex regulatory cycles which are dysregulated in the case of Alzheimer's disease.

Sphingolipids are a heterogeneous group of lipids, structurally based on the 18-carbon amino alcohol sphingosine which is synthesized from palmitoyl-CoA and serine by serine palmitoyl-CoA transferase (SPT), representing the rate-limiting step in sphingolipid synthesis and shown to be regulated by AICD [122]. Ceramide (Cer) is an important branching point for the synthesis of different sphingolipid subspecies. For example, sphingomyelin (SM) is generated out of Cer by SM-synthase, whereas the neutral sphingomyelinase (nSMase) catalyzes the turnover of SM to Cer. Glycosylation of Cer results in the production of glycosphingolipids which can be further processed to gangliosides. The cerebroside synthase adds a galactose moiety to Cer which is the first step towards the formation of sulfatides. Finally, the decomposition of Cer is provided by the ceramidase receiving sphingosine which itself can be phosphorylated to sphingosine-1-phosphate (S1P). First evidence of sphingolipid metabolism being involved in neurodegenerative diseases was derived from investigations of lysosomal storage diseases (LSDs). This group of inherited metabolic disorders is characterized by accumulation of different sphingolipids due to dysfunction or deficiency of the corresponding lysosomal enzymes. Importantly, affected patients clinically develop progressive cognitive decline resulting in early dementia. Additionally, AD-related pathologies like A*β* accumulation and hyperphosphorylation of tau, leading to neurofibrillary tangles, can be observed [123, 124]. Beside these similarities between AD and LSD, several studies of AD *postmortem* brains indicate that the sphingolipid metabolism is altered during AD progression, further substantiated by biochemical studies, linking sphingolipids to APP processing [125–131] (Figure 2). 

### 4.1. Ceramide

The majority of *postmortem* brain tissue analysis found elevated Cer levels in the grey and white matters of AD patients. These alterations were observed even in early stages of AD hypothesizing that these might promote the development of the disease [125, 128, 131, 132]. In line with these findings, gene expression abnormalities of the key enzymes that control sphingolipid metabolism were found in AD patients: enzymes involved in glycosphingolipid synthesis (e.g., UDP-glucose ceramide glucosyltransferase) were altered accompanied by changes in enzymes resulting in the accumulation of Cer (e.g., serine palmitoyltransferase, neutral sphingomyelinase, and acid sphingomyelinase) [131, 133]. Recently, Mielke et al. reported in a 9-year-follow-up study that even elevated baseline serum Cer levels are associated with a higher risk (up to 10-fold) of developing AD [134], indicating that serum Cer is associated with incident AD. 

In contrast to the results obtained from AD *postmortem* brains, the analysis of AD mouse models revealed inconsistent data. Cer levels are elevated in the cerebral cortex of APP^SL^/PS1Ki transgenic mice, whereas the corresponding single-transgenic mice did not show this alteration [135]. Furthermore, in APP^SL^/PS1^M146L^ transgenic mice, in which the time course of pathology is closer to that seen in most currently available models, Cer did not accumulate in disease-associated brain regions (cortex and hippocampus) [136]. These different findings might be attributed to the various mouse models used in these studies [135, 136]. The authors suggest that APP^SL^/PS1Ki mice, compared to the other mouse models, produce exceeding amounts of N-truncated A*β*x-42, also found in AD brains [135].

It is well established that ceramides induce apoptosis and exhibit neurotoxic properties [137–139]. Interestingly, A*β* toxicity is linked to Cer-dependent apoptotic pathways. Lee et al. observed an A*β*-induced elevation of nSMase and consequently increased Cer levels which resulted in remarkable cell death. Inhibiting this pathway abolished the A*β*-triggered cascade [140]. In this context, activation of nSMase by A*β*42 but not A*β*40 has been reported [66, 141] (Figure 2). Recently, a potential novel mechanism of ceramide-enriched exosomes released by A*β*-treated astrocytes was proposed to be responsible for A*β*-induced apoptosis. Thereby, nSMase2 was essential for charging these exosomes with Cer [142, 143]. Beside the involvement of Cer in A*β* toxicity, Cer has been shown to alter APP processing and A*β* production. Increasing Cer levels by either direct Cer administration or stimulation of endogenous biosynthesis by nSMase resulted in enhanced A*β* production. Elevated A*β* levels are attributed to Cer-induced protein stabilization of *β*-secretase BACE1, whereas *γ*-secretase is not affected [144]. Further studies elucidated the underlying mechanism of increased BACE1 stability: elevated Cer level caused upregulation of the acetyltransferases, ATase1, and ATase2, acetylating BACE1 protein and thus protecting the nascent protein from degradation [145]. Taken together, it seems likely that Cer is the driving force in a circulus vitiosus: increasing Cer levels lead to an intensified A*β* production whereupon A*β* is responsible for Cer accumulation.

Noteworthy in this context, S1P is discussed as a possible counterpart. S1P is considered to be neuroprotective and important for neuronal differentiation [146, 147]. Remarkably, one study reported reduced S1P levels in frontotemporal grey matter of AD patients [131], which implicates a possible role in AD. However, recent findings reported an increased proteolytic activity of BACE1 by direct interaction with S1P [148]. Therefore, additional studies addressing S1P are important to clarify the significance of S1P in APP processing and AD.

### 4.2. Sphingomyelin

Sphingomyelin is an important component of mammalian cell membranes, particularly enriched in myelin sheets and especially represented in the brain [149]. Considering the observations outlined in the preceding chapter it is suggested that SM concentrations in AD brains might be decreased. Analysis of *postmortem* brain, however, show inconsistent results. Although two studies reported elevated SM levels in AD brains compared to age-related controls [150, 151], two studies described a significant decrease [131, 132]. Further studies only revealed a modest reduction in severe AD *postmortem* brains and no significant differences in earlier stages of the disease [125]. Moreover, recent investigations found increased SM levels in the cerebrospinal fluid (CSF) of individuals suffering from prodromal AD, whereas there was only a slight but not significant decrease in mild and moderate AD groups [152]. Interestingly, a current epidemiological study correlated higher plasma SM levels with slower cognitive decline among AD patients, illustrating SM as potential sensitive blood-based biomarker for disease progression [153]. Importantly, nSMase was reported to be upregulated in AD brains [133], resulting in increased SM breakdown. Additionally, in cell culture studies, nSMase activity is known to be elevated in presenilin familial Alzheimer's disease mutations (PS-FAD) causing early onset AD [66], pointing towards a possible role of SMases in sporadic late onset as well as in familial early onset AD pathology. Interestingly, SM itself is discussed to alter APP processing. Increasing SM by either direct treatment of cells with SM or inhibition of nSMase resulted in diminished A*β* levels [66]. In this context, it is again worth mentioning that A*β*42 itself regulates SM homeostasis as described previously.

### 4.3. Gangliosides

Gangliosides are a family of sialic acid containing glycosphingolipids, highly expressed in neuronal and glial membranes, where they play important roles for development, proliferation, differentiation, and maintenance of neuronal tissues and cells [154, 155]. The first step towards the formation of gangliosides is the glycosylation of Cer by glucosylceramide-synthase (GCS). According to their number of sialic acid residues, gangliosides are separated in four different ganglio-series: *o*-series, *a*-series, *b*-series, and *c*-series. Importantly, the most common brain gangliosides belong either to the *a*-series (GM1 and GD1a) or *b*-series (GD1b and GT1b). The ganglioside GM3 serves as a common precursor for *a*- and *b*-series gangliosides. The GD3-synthase (GD3S) catalyzes the synthesis of GD3 by adding sialic acid to GM3, segregating the *a*- and *b*-series of gangliosides [156] and therefore controlling the levels of the major brain gangliosides. Gangliosides are also able to interact with further membrane lipids like SM and cholesterol, thereby, being involved in the formation of lipid rafts [157]. Interestingly, *postmortem *studies of AD brains suggest a strong connection between ganglioside homeostasis and AD pathology. In a previous work, Kracun et al. found all major brain gangliosides to be reduced in the temporal and frontal cortex and in nucleus basalis of Meynert, whereas simple gangliosides GM2 and GM3 were elevated in parietal and frontal cortex [158]. GM3 elevation was further supported by a recent study. Here, the authors also described an increase in glucosylceramide levels, the precursor for ganglioside synthesis [159]. Additionally, Gottfries et al. reported a significant reduction of gangliosides in the grey matter of early onset AD subjects compared to late onset AD and control individuals. Nevertheless, a decrease in total gangliosides was also observed in brains of late onset AD patients, however, to a smaller extent [160]. A more recent study found elevated levels of GM1 and GM2 in lipid raft fractions of the temporal and frontal cortex of AD brains [161]. Moreover, the analysis of AD transgenic mouse models suggests an altered ganglioside metabolism in AD. Barrier et al. compared different transgenic mice with age-matched wildtype controls. While all mice expressing APP (SL) showed an increase in GM2 and GM3 in the cerebral cortex and a moderate decrease in complex *b*-series gangliosides, only APP/PS1Ki transgenic mice exhibited a loss of complex* a*-series gangliosides, GT1a, GD1a, and GM1 [162]. In summary, ganglioside metabolism seems to be highly affected during disease progression. While more complex gangliosides appear to be depleted, simple gangliosides, like GM1 and GM3 are increased. As mentioned previously GM3 is an important precursor for *a*- and *b*-series gangliosides suggesting a disease-dependent alteration in the biosynthesis of these ganglioside series. Indeed, we found a close link between APP processing products and ganglioside metabolism. The activity of GD3S, the key enzyme converting *a*- to *b*-series gangliosides, is significantly reduced by two separate and additive mechanisms. On one hand, GD3S activity is inhibited by the binding of A*β* peptides to GM3, consequently reducing substrate availability and preventing the conversion of GM3 to GD3. On the other hand, gene transcription of GD3S is downregulated and mediated by the APP intracellular domain (AICD), thus, resulting in GD3 depletion and GM3 accumulation [163]. 

Furthermore, especially GM1 is related to several AD-specific pathomechanisms like altered APP processing, aggregation, and cytotoxicity. GM1 was found to decrease sAPP*α* levels and to increase A*β* generation, whereas sAPP*β* levels were unchanged [164]. This suggests increased *γ*-secretase and decreased *α*-secretase activities without affecting *β*-secretase. Indeed, as recently described, the direct administration of gangliosides to purified *γ*-secretase resulted in an increased enzyme activity. Moreover, a shift towards the formation of A*β*42 peptides was observed [165]. Interestingly, A*β* decreases membrane fluidity by binding GM1. As a consequence, amyloidogenic APP processing was stimulated, proposing an A*β*-triggered, GM1-mediated, vicious cycle [166]. Further mechanisms linking gangliosides to A*β* production were described by Tamboli et al. Inhibition of GCS, the committed step towards ganglioside formation, significantly decreased A*β* formation. An altered APP maturation and cell surface transport, leading to less access of APP to amyloidogenic processing in the endosomal compartments, were proposed as the underlying mechanisms [167]. 

Furthermore, GM1 seems to be particularly important as a “seed” for amyloid plaque formation. Thereby, GM1 interacts with A*β*, resulting in GA*β* complexes [168]. This complex tends to aggregate more easily due to changing the secondary structure of A*β* towards *β*-sheet formation [169, 170]. Interestingly, Mahfoud et al. described a sphingolipid binding domain of A*β*, which is also contained in HIV-1 and prion proteins [171]. Further investigations displayed accumulation and aggregation of A*β* on cell membranes especially in GM1-enriched lipid rafts, resulting in enhanced cytotoxicity [172, 173]. Additionally, enhanced cytotoxicity of A*β* fibrils was observed after release of GM1 from damaged neurons, indicating a possible aggravation mechanism [174]. Importantly, GA*β* complexes also occur in AD brains and aged mice [175]. Synaptosomes, prepared from aged mouse brains, exhibited GM1 clusters, showing high ability to initiate A*β* aggregation [176]. Remarkably, A*β* deposition was observed to begin mainly at the presynaptic neuronal membranes in AD brains, suggesting a possible role of GA*β* in the early pathogenesis of AD [177, 178].

### 4.4. Sulfatides

Another sphingolipid subgroup, possibly involved in AD pathogenesis, are sulfatides. They are generated from Cer by adding a galactose moiety, catalyzed by the ceramide galactosyltransferase (CGT). Finally, synthesis of sulfatides is provided by cerebroside sulfotransferase (CST), which transfers a sulfate group to the galactosyl moiety. The degradation of sulfatides takes place in the lysosomal compartment, where arylsulfatase A (ASA) hydrolyzes the sulfate group. ASA deficiency leads to accumulation of sulfatides and to the clinical picture of metachromatic leukodystrophy. Sulfatides are especially enriched in myelin sheaths making up about 5% of the myelin lipids. Hence, they are particularly produced by oligodendrocytes and Schwann cells. Nevertheless, sulfatides have also been detected in neurons and astrocytes, however, in a lower amount [179]. Interestingly, AD pathology is known to induce focal demyelination and degeneration of oligodendrocytes [180].

Importantly, Han et al. described an extraordinary depletion of sulfatide levels in all analyzed brain regions of AD subjects. Sulfatides were depleted up to 93% in grey matter and up to 58% in white matter. These alterations were already observed in the earliest stages of the disease [125]. Additionally, a previous study found decreased sulfatide levels in the white matter of the frontal lobe only in patients with late onset AD compared to age-matched controls and early onset AD patients [160]. Furthermore, Bandaru et al. also described decreased sulfatide content in the white matter; however, they did not confirm the alterations in the grey matter [150]. In contrast, further studies found no significant changes between control and AD brains [132, 159]. Worth mentioning, a 40% reduction in CSF sulfatide levels was detected among AD patients. In this context, sulfatide/phosphatidylinositol ratio was proposed as potential clinical biomarker for early AD diagnosis [181]. However, data obtained by analyzing transgenic mouse models in respect to alterations in sulfatide levels are more inconsistent. Although Barrier et al. found no significant changes between wildtype mice and double transgenic APP^SL^/PS1Ki mice or single-transgenic PS1Ki mice [135], a more recent study found significantly decreased sulfatide levels in the forebrain of these mouse models [159]. 

Noteworthy, there is a possible link between sulfatide homeostasis and ApoE trafficking. First, the intercellular sulfatide transport in the brain is mediated by ApoE-containing lipoproteins. Interestingly, human ApoE4 carrying transgenic mice presented the highest sulfatide depletion in the brain in comparison to wildtype ApoE or human ApoE3 transgenic mice [181]. Furthermore, a recent study showed a significant, age-related decrease of sulfatides in APP transgenic mice, which was completely abolished by ApoE knockout [182]. These findings offer a possible explanation for decreased sulfatide levels in AD brains. On the other hand, sulfatides seem to be involved in the ApoE-dependent A*β* clearance. Direct supplementation of sulfatides to cultured cells dramatically reduced A*β* levels. This observation was most likely ascribed to a modification of A*β* clearance through an endocytotic pathway. Thereby, sulfatides facilitated the ApoE-mediated A*β* clearance, especially of ApoE4 containing vesicles [183]. Nevertheless, the importance of sulfatide-dependent molecular mechanisms, being involved in AD pathogenesis, is still ambiguous. Therefore, further investigations are necessary to clarify the role of sulfatides in APP processing and AD. 

## 5. Lipids and Tau Pathology

Beside amyloid plaques, neurofibrillary tangles (NFTs), consisting of hyperphosphorylated tau proteins, are considered to be a pivotal pathological hallmark of AD [184–186]. Although this review focuses on the impact of lipids on APP processing, it is worth mentioning that tau pathology is also affected by an altered lipid homeostasis. Tau proteins belong to the family of microtubule-associated proteins, important for the assembly of tubulin monomers into microtubules, to stabilize the neuronal microtubule network which is essential for maintaining cell shape and axonal transport [187]. In AD, this function is disrupted, due to the hyperphosphorylation of serine/threonine residues of tau. This abnormal phosphorylation promotes the release of tau proteins from microtubules, its disassembly, and self-assembly into paired helical filaments (PHFs), a major component of NFTs, and, as a consequence, provokes microtubule disruption [188–191]. Supporting the connection between lipids and tau, Kawarabayashi et al. reported an accumulation of phosphorylated tau in brain-extracted lipid rafts of an AD mouse model [192]. In addition, tau phosphorylation by cyclin-dependent kinase 5 (CDK5) was observed in lipid rafts after short-term incubation of SH-SY5Y cells with A*β* peptides [193]. Interestingly, Niemann-Pick type C (NPC), an inherited lysosomal storage disease with an abnormal intracellular accumulation of cholesterol, also exhibits tau pathology [194, 195]. Noteworthy, these alterations are more pronounced in neurons containing higher cholesterol levels [194, 196]. In line with these findings elevated A*β* and total tau levels were observed in the cerebrospinal fluid of NPC patients [197]. In an NPC mouse model, Sawamura et al. illustrated a possible explanation for these hyperphosphorylated tau forms. They found an intensified activation of the mitogen-activated protein kinase (MAPK)-pathway, one of several kinases phosphorylating tau physiologically in the brain [198]. Not only in NPC model mice but also in further mouse models, the cholesterol and ApoE status were associated with increased tau phosphorylation [199–201]. In contrast, statin treatment reduced NFT burden in normocholesterolemic and hypercholesterolemic mice. This effect, however, was rather attributed to the anti-inflammatory properties than to the cholesterol lowering aspect of statins [202]. Additionally, simvastatin treatment of hypercholesterolemic subjects without dementia revealed a significant phospho-tau-181 decrease in the CSF, whereas no differences in total tau or A*β* levels were observed [203]. Importantly, membrane cholesterol levels are closely linked to the A*β*-induced calpain activation and tau toxicity [204]. Calpain is a calcium-dependent cysteine protease responsible for the generation of the neurotoxic 17 kDa tau [205, 206]. Decreasing membrane cholesterol in mature neurons reduced their susceptibility to the A*β*-induced calpain activation, 17 kDa tau production, and cell death, whereas elevated membrane cholesterol levels enhanced this A*β*-triggered cascade in young neurons [204]. Like calpain, AMP-activated protein kinase (AMPK), a serine/threonine kinase, is also activated by elevated calcium levels. Interestingly, in addition to its important function in regulating cholesterol homeostasis, emerging studies revealed AMPK as a potential tau phosphorylating enzyme [207, 208]. AMPK-induced abnormal tau phosphorylation inhibited microtubule binding of tau [207]. In line with these findings Vingtdeux et al. reported an accumulation of activated AMPK in cerebral neurons of AD brains [209]. Other authors, however, even ascribed AMPK an inhibiting function in tau phosphorylation by downregulating glycogen synthase kinase-3*β* (GSK3*β*) activity, one of the main tau phosphorylating kinases [210, 211]. Beside cholesterol, *ω*-3 fatty acids are also discussed to influence tau pathology. In a 3xTg-AD mouse model, Green et al. demonstrated lowered levels of intraneuronal tau and reduced tau phosphorylation after 3 to 9 months of DHA supplementation [112]. Similar results were also observed by Ma et al. after fish oil administration to 3xTg-AD mice [212]. In both studies, the reduced phosphorylation was attributed to an inhibition of the c-Jun N-terminal kinase (JNK). In contrast, low *ω*3 intake with a decreased *ω*3 : *ω*6 ratio leads to an aggravation of tau pathology in these transgenic mice [213]. Furthermore, some studies revealed a colocalization of sphingolipids and gangliosides with PHFs proposing a possible relation between sphingolipid metabolism and tau [214, 215]. Indeed, inhibition of the serine palmitoyl transferase (SPT), the rate-limiting enzyme in sphingolipid synthesis, and the first step towards ceramide synthesis resulted in a reduced tau hyperphosphorylation in an AD mouse model [216]. After treatment of differentiated PC12 cells with ceramide derivates (N-acetylsphingosine, and N-hexanoylsphingosine), Xie and Johnson reported a significant reduction in tau levels without affecting tau phosphorylation. This was attributed to an increased expression of calpain I and thus stimulated tau protein degradation [217]. Taken together, not only APP processing but also tau pathology is influenced by lipids. Although the general view for APP processing seems more consistent, further investigations linking tau to altered lipid homeostasis should follow.

## 6. Conclusion

Summing it up, lipids are tightly linked to AD. It has been shown that cholesterol increases amyloidogenic pathways and decreases non amyloidogenic pathways followed by an enhanced A*β* production and aggregation. Opposite effects were observed for DHA, suggesting a potential beneficial role for DHA or PUFAs in AD. For sphingolipids and glycosphingolipids, a more complex situation in respect to AD is reported. Although lipids like SM, S1P, and sulfatides seem to be protective by enhancing A*β* clearance or decreasing A*β* production, other glycosphingolipids like gangliosides or ceramides increase A*β* toxicity or A*β* oligomerization. Interestingly, it has been shown that, in return, APP processing also affects lipid metabolism, resulting in complex regulatory feed-back cycles, which seem to be dysregulated in AD. In line, several studies suggest an altered lipid metabolism in human AD brains. However, controversial effects are reported in different brain regions and tissues, making more detailed analysis with new lipidomic approaches and higher numbers necessary.

## Supplementary Material

Supplementary material provides a tabular overview of the proposed mechanisms of cholesterol on APP processing with selected publications included.

## Figures and Tables

**Table 1a:** (a) Effect of DHA

Affected pathway	Mechanism of action
Nonamyloidogenic processing	sAPP*α* ↑
	ADAM 17 protein stability ↑

Amyloidogenic processing	A*β*↓
	*β*-Secretase activity ↓
	Endosomal BACE1 ↓
	*γ*-Secretase activity ↓
	PS1 shift: raft → non-raft

A*β* Oligomerization and toxicity	A*β* Fibrillation ↓
	Soluble toxic oligomers ↓
	A*β* Phagocytosis ↑

Cholesterol homeostasis	HMG-CoA reductase activity ↓
	Cholesterol *de novo* synthesis ↓
	Cholesterol shift: raft → non-raft

Other non-APP-mediated pathways/mechanisms	SorLA/R11 ↑, a sorting protein reduced in AD
	Neuronal differentiation ↑
	Protection against synaptic loss, Synaptogenesis ↑
	Neurogenesis ↑
	Inflammation ↓
	Reactive oxidative species ↓


**Table 1b:** (b) Effect of DHA derivates

Affected pathway		Mechanism of action
NPD1	Nonamyloidogenic processing	sAPP*α* ↑
		ADAM 10 maturation ↑
	Amyloidogenic processing	A*β*↓
		sAPP*β*↓
		BACE 1 protein level ↓
	A*β* Toxicity	Neuroprotective and antiapoptotic
		Soluble toxic oligomers ↓
