# Supplementary material for: The Impact of Cholesterol, DHA, and Sphingolipids on Alzheimer's Disease
Source: Biomed Res Int. 2014 Feb 19;2013:814390. doi: 10.1155/2013/814390 (PMC3929518; doi:10.1155/2013/814390)
Supplement: Supplementary file 1 — Supplementary material provides a tabular overview of the proposed mechanisms of cholesterol on APP processing with selected publications included. [file 814390.f1.docx]

**Supplementary material**

**Supplementary Table 1**: Overview of proposed mechanisms of cholesterol on APP processing including selected publications. Cholesterol has been shown to increase amyloidogenic and to decrease non-amyloidogenic APP processing leading to an enhanced Aβ production by pleiotropic mechanisms further accelerating AD pathology by affecting Aβ toxicity, aggregation and clearance. Epidemiological studies or clinical trials are not summarized here but are reviewed e.g. in [1-2] or [3].

Supplementary Table 1:

| **Effect of cholesterol on Aβ production :** | | |  |
| --- | --- | --- | --- |
| **Mechanism of action / key findings** | **Experimental approach** | **Selected literature** |  |
| ↓ Aβ production | cholesterol depletion in hippocampal neurons | [4] |  |
| ↑ Aβ deposition, ↑ AD pathology | hypercholesterolemia in transgenic mouse model | [5] |  |
| ↓ Aβ peptides, ↓ Aβ load | cholesterol-lowering drug BM15.766 in transgenic AD mouse model | [6] |  |
| ↓ Aβ40&Aβ42 | cholesterol depletion by simvastatin treatment of guinea pigs and cell culture experiments | [7] |  |
|  |  |  |  |
| **Effect of cholesterol on β- and γ-secretase activity:** | | |  |
| **Mechanism of action / key findings** | **Experimental approach** | **Selected literature** |  |
| ↓ β- & γ-secretase activity | cholesterol depletion by statins and cyclodextrin (CDX) in SH-SY5Y cells | [8] |  |
| ↑ β- & γ -secretase activity | addition of cholesterol on human AD samples and N2A cells | [9] |  |
| ↓ β-secretase activity | measurement of β-secretase activity of recombinant full length BACE1 in reconstituted vesicles supplemented with cholesterol | [10] |  |
| positive correlations of β-secretase activity and cholesterol at higher membrane cholesterol levels | membrane β-secretase activity in the presence of a range of membrane cholesterol levels in SH-SY5Y human neuroblastoma cells after cholesterol addition | [11] |  |
| ↓ γ -secretase activity | measurement of γ -secretase activity in buoyant membrane fractions containing presenilin 1 after cholesterol depletion | [12] |  |
| **Effect of cholesterol on BACE1:** | | |  |
| **Mechanism of action / key findings** | **Experimental approach** | **Selected literature** |  |
| ↓ APP/BACE proximity | FRET based proximity measurement of APP and BACE1 after cellular cholesterol depletion | [13] |  |
| ↓ BACE dimerization | statins added in cell culture experiments | [14] |  |
| ↑ BACE level, | feeding rabbits with 1% cholesterol for 7 months | [15] |  |
| **Effect of cholesterol on non amyloidogenic processing:** | | |  |
| **Mechanism of action / key findings** | **Experimental approach** | **Selected literature** |  |
| ↓ sAPPα | cholesterol addition to HEK293 cells transfected with APP695 | [16] |  |
| ↑ non-amyloidogenic pathway,  ↑ expression of ADAM10 | reduction of cholesterol in peripheral and neuronal cell lines by treatment with CDX or statins | [17] |  |
| ↓ sAPPα, ↑detergent soluble Aβ | high cholesterol diet in transgenic mice for 6 weeks | [18] |  |
| **Effect of cholesterol on gene expression of genes involved in amyloidogenic processing:** | | |  |
| **Mechanism of action / key findings** | **Experimental approach** | **Selected literature** |  |
| ↑ PS1 and PS2 expression | cells exposed to cholesterol | [19] |  |
| **Effect of cholesterol on Aβ aggregation and clearance:** | | |  |
| **Mechanism of action / key findings** | **Experimental approach** | **Selected literature** | |
| cholesterol acts as a promoter for Aβ-membrane interactions facilitating Aβ aggregation | effect of cholesterol levels on interaction of Aβ(1-42) monomer with 1-palmitoyl-2-oleoylphosphatidylcholine bilayer using all-atom molecular dynamics simulations | [20] | |
| cholesterol alters membrane-Aβ interactions via fine tuning of glycosphingolipid conformation | molecular modeling simulations and surface pressure measurements of membranes | [21] | |
| Aβ-cell surface interactions are mediated by cellular cholesterol levels | fluorescence microscopy studies of PC-12 and SH-SY5Y,  situ scanning probe microscopy, fluorescence anisotropy, and electron microscopy to investigate within brain lipid bilayers | [22] | |
| Aβ(1-42) preferentially binds to cholesterol-rich domains of cell membranes and forms amyloidosis | interaction of native Aβ(1-42) with PC12 cells was visualized using Congo red | [23] | |
| derivates of cholesterol induce Aβ aggregation, generation of reactive oxygen species and cytotoxicity | addition of cholesterol derivates to murine GT1-7 hypothalamic neurons | [24] | |
| ↓ insoluble Aβ, Aβ-oligomers from AD brains associate with raft membrane fraction in a cholesterol-dependent manner | cholesterol depletion in neuronal cells, analysis of human *post mortem* AD samples | [25] | |
| **Effect of cholesterol on Aβ & ROS:** | | |  |
| **Mechanism of action / key findings** | **Experimental approach** | **Selected literature** |  |
| Aβ promotes oxidative stress directly by catalytically producing H_2_O_2_ from cholesterol | effect of high cholesterol on neuroblastoma cells in Aβ-mediated neurotoxicity | [26] |  |

| **Effect of cholesterol distributions / Lessons from NPC models:** | | |  |
| --- | --- | --- | --- |
| **Mechanism of action / key findings** | **Experimental approach** | **Selected literature** |  |
| ↑ β-CTF, ↑ γ-secretase activity, ↑Aβ40&Aβ42, abnormal distribution of PS1 in the endosomal system | mouse model of Niemann-Pick type C disease; in these mouse brains, cholesterol accumulates in late endosomes/ lysosomes | [27] |  |
| ↑ γ-secretase activity on APP C-terminal fragments, ↑Aβ40&Aβ42, PS1 and PS2 accumulation in Rab7-positive vesicular organelles | addition of cholesterol transport-inhibiting agents in neuronal and CHO cells and analysis of NPC1 deficient cells | [28] |  |
| ↑ Aβ accumulation in late endosomes | Niemann-Pick type C (NPC) model cells and NPC mutant cells, showing aberrant cholesterol trafficking, & NPC mouse brain | [29] |  |
| ↓ expression of APP at the cell surface and ↑ processing of APP through the β-secretase pathway resulting in ↑ β-CTF, sAPPβ and intracellular Aβ40 levels | analysis of NPC1 deficient cells | [30] |  |
| shift in fl-APP/CTF compartmentalization into lipid raft fractions | raft fractions of CHO NPC1(-/-) cells (NPC cells) and parental CHOwt cells were analyzed | [31] |  |
| **Effect of cholesterolesthers and ACAT:** | | | |
| **Mechanism of action / key findings** | **Experimental approach** | **Selected literature** |  |
| cholesteryl-ester levels directly correlate with Aβ production | genetic, biochemical and metabolic approaches altering the choleterolesthers in different cells | [32] |  |
| ↓ cognitive deficits, ↓ APP and APP proteolytic fragments | analysis of ACAT1 gene ablation in triple transgenic AD mice | [33] |  |
